# Phylogeny of hydrothermal vent Iphionidae, with the description of a new species (Aphroditiformia, Annelida)

**DOI:** 10.3897/zookeys.779.24781

**Published:** 2018-08-02

**Authors:** Marina F. McCowin, Greg W. Rouse

**Affiliations:** 1 Scripps Institution of Oceanography, University of California San Diego, La Jolla, CA 92093-0202, USA University of California San Diego La Jolla United States of America

**Keywords:** East Pacific Rise, Pacific Ocean, polychaete, systematics, scale-worm

## Abstract

The scale-worm family Iphionidae consists of four genera. Of these, *Thermiphione* has two accepted species, both native to hydrothermal vents in the Pacific Ocean; *T.fijiensis* Miura, 1994 (West Pacific) and *T.tufari* Hartmann-Schröder, 1992 (East Pacific Rise). *Iphionella* is also known from the Pacific, and has two recognized species; *Iphionellarisensis* Pettibone, 1986 (East Pacific Rise, hydrothermal vents) and *I.philippinensis* Pettibone, 1986 (West Pacific, deep sea). In this study, phylogenetic analyses of Iphionidae from various hydrothermal vent systems of the Pacific Ocean were conducted utilizing morphology and mitochondrial (COI and 16S rRNA) and nuclear (18S and 28S rRNA) genes. The results revealed a new iphionid species, described here as *Thermiphionerapanui***sp. n.** The analyses also demonstrated the paraphyly of *Thermiphione*, requiring *Iphionellarisensis* to be referred to the genus, as *Thermiphionerisensis* (Pettibone, 1986).

## Introduction

Annelid scale-worms (Aphroditiformia) are a particularly common and diverse group at hydrothermal vents (Desbruyères et al. 2006). Most of this diversity is within Polynoidae Kinberg, 1856, but there have been several records of another aphroditiform family, Iphionidae Kinberg, 1856, which currently includes four genera and 13 accepted species (Read and Fauchald 2018). Iphionidae had been regarded as a subfamily of Polynoidae, until Norlinder et al. (2014) gave it family rank, as it appears it is actually most closely related to Acoetidae (Gonzalez et al. 2018). In addition to DNA sequence data, the monophyly of Iphionidae is supported by the presence of feathered notochaetae, areolae on elytra, and the absence of a median antenna (Gonzalez et al. 2018). The majority of the known diversity of iphionids are within *Iphione* Kinberg, 1856, and these are mostly shallow-water taxa. However, three genera of deep-sea hydrothermal vent iphionids have been described: *Iphionella* McIntosh, 1885 and *Thermiphione* Hartmann-Schröder, 1992, each with two species, and *Iphionides* Hartmann-Schröder, 1977, containing only *I.glabra* Hartmann-Schröder, 1977.

With regards to the hydrothermal vent-associated iphionids, *Iphionellarisensis* Pettibone, 1986 was erected for specimens collected from the East Pacific Rise at 20°50'N. Similar to *I.philippinensis*, this species has 13 pairs of elytra. *Thermiphionetufari* Hartmann-Schröder, 1992, was described for specimens also collected from the East Pacific Rise at 21°30'S, well to the south of the type locality of *I.risensis*. A new genus, *Thermiphione* Hartmann-Schröder, 1992, was erected for this species. *Thermiphione* was distinguished from *Iphionella* by the presence of 14 pairs of elytra instead of 13, as well as by having a greater number of segments (Hartmann-Schröder 1992). *Thermiphionefijiensis* Miura, 1994 was subsequently described from hydrothermal vents from the western Pacific (North Fiji Basin), also with 14 pairs of elytra (Miura 1994).

This paper focuses on new deep-sea collections of Iphionidae from Pacific Ocean hydrothermal vents. DNA data was previously published for *Thermiphionefijiensis* (as *Thermiphione* sp.) in Norlinder et al. (2012); herein we add additional DNA data for this species and for the other two known hydrothermal vent Iphionidae. Furthermore, we describe a new vent-associated iphionid species from the East Pacific Rise and assess some morphological and taxonomic issues for Iphionidae.

## Materials and methods

### Sample collection

**Figure 1.**
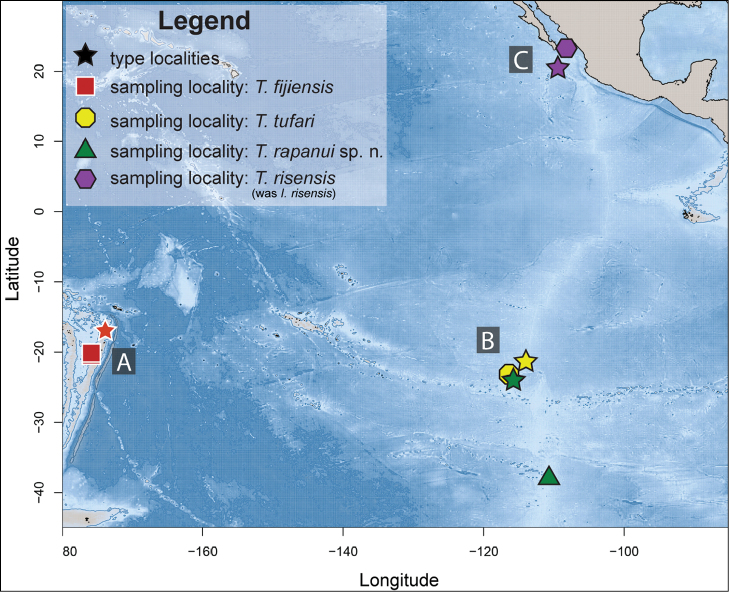
Map of sampling localities for iphionids in this study. Species differentiated by color and shape, type localities represented by stars. **A***Thermiphionefijiensis* type (star) and sampling (square) localities **B***Thermiphionetufari* type (star) and sampling (octagon) localities, as well as *Thermiphionerapanui* sp. n. localities (triangle) **C***Thermiphionerisensis* (was *Iphionellarisensis*) type (star) and sampling (hexagon) localities.

Sampling was conducted over several years and at multiple localities (Figure 1, Tables 1, 2). *Thermiphionerapanui* sp. n. and *T.tufari* were collected on several dives by the manned submersible *Alvin* in 2005 at hydrothermal vents of the southern East Pacific Rise (Table 2). *Thermiphionefijiensis* was collected from the Lau Back-arc Basin in 2005 utilizing the ROV *Jason II* (Table 2). *Iphionellarisensis* was collected in 2012 using the ROV *Doc Ricketts* from the Alarcon Rise in the Gulf of California, just north of its type locality (Table 2). All specimens are deposited in the Scripps Institution of Oceanography Benthic Invertebrate Collection (SIO-BIC), La Jolla, California, USA. Whole specimens were photographed prior to preservation using Leica MZ8 or MZ9.5 stereomicroscopes. Post-preservation, specimens were examined and photographed using Leica S8 APO and DMR HC microscopes.

**Table 1.** Origin of sequenced terminals, vouchers, and GenBank accession numbers. New sequences in bold. Family assignments follow Zhang et al. (2018).

Scientific name	Origin	Voucher	18S	28S	16S	COI
* Panthalis oerstedi *	Sweden	SMNH118954	AY839572	JN852845	JN852881	AY839584
Iphione cf. treadwelli	Eilat, Israel	–	KY823447	–	KY823478	KY823494
*Iphione* sp. 1	Hong Kong	–	KY753852	KY753852	KY753835	KY753835
*Iphione* sp. 2	Papua New Guinea	SMNH118972	JN852819	–	JN852886	JN852921
***Iphione* sp. 3**	Lord Howe Island, Australia	SIO-BIC A8708	–	–	–	**MH389786**
***Thermiphionerisensis*** (was *Iphionellarisensis)*	Gulf of California	SIO-BIC A6326	**MG994954**	**MH000396**	**MG994947**	**MG981037**
*** Thermiphione tufari ***	East Pacific Rise	SIO-BIC A7973	**MG994958**	**MH000401**	**MG994951**	**MG981042**
*Thermiphione* sp. *(fijiensis)*	Fiji, Lau Basin	SMNH118982	JN852820	JN852849	JN852887	JN852922
*** Thermiphione fijiensis ***	Lau back-arc Basin	SIO-BIC A7975	**MG994960**	**MH000402**	**MG994953**	**MG981044**
***Thermiphionerapanui* sp. n.**	East Pacific Rise	SIO-BIC A7969	**MG994955**	**MH000397**	**MG994948**	**MG981038**

**Table 2.** Sampling localities and GenBank COI accession numbers for all specimens collected and sequenced for this study.

Specimen	Voucher	Locality	Latitude / Longitude	Depth (m)	COI Accession No.
* Iphionella risensis *	SIO-BIC A6326	Alarcon Rise, Gulf of California	23°22'37"N, 108°31'52"W	2,309	MG981037
*Thermiphionerapanui* sp. n.	SIO-BIC A7969	Pacific Antarctic Ridge	37°47'60"S, 110°55'0"W	2,216	MG981038
*Thermiphionerapanui* sp. n.	SIO-BIC A7970	Pacific Antarctic Ridge	37°47'60"S, 110°55'0"W	2,216	MG981039
*Thermiphionerapanui* sp. n.	SIO-BIC A8557	Pacific Antarctic Ridge	37°47'60"S, 110°55'0"W	2,216	–
*Thermiphionerapanui* sp. n.	SIO-BIC A7971	East Pacific Rise	23°32'47"S, 115°34'11"W	2,595	MG981040
*Thermiphionerapanui* sp. n.	SIO-BIC A7972	East Pacific Rise	23°32'47"S, 115°34'11"W	2,595	MG981041
* Thermiphione tufari *	SIO-BIC A7973	East Pacific Rise	23°32'47"S, 115°34'11"W	2.595	MG981042
* Thermiphione tufari *	SIO-BIC A7974	East Pacific Rise	23°32'47"S, 115°34'11"W	2.595	MG981043
* Thermiphione fijiensis *	SIO-BIC A7975	Lau Back-Arc Basin	20°19'0"S, 176°9'0"W	2,719	MG981044
* Thermiphione fijiensis *	SIO-BIC A8510	Kilo Moana, Lau Back-Arc Basin	20°3'0"S, 176°9'0"W	2,657	MG981045
*Iphione* sp. 3	SIO-BIC A8708	Lord Howe Island, Australia	31°31.603'S, 159°4.518'E	5	MH389786

### DNA extraction and amplification

DNA extraction of specimens from the aforementioned collection sites was conducted with the Zymo Research DNA-Tissue Miniprep kit, following the protocol supplied by the manufacturer. Up to 645 bp of mitochondrial cytochrome subunit I (COI) were amplified using the primer set HCO2198 and LCO1490 (Folmer et al. 1994) for multiple specimens in Table 2 and 16S rRNA, 18S rRNA, and 28S rRNA were amplified for a subset of these specimens. Up to 527 bp of 16S rRNA (16S) were amplified using the primer set 16SbrH and 16SarL (Palumbi 1996). 18S rRNA was amplified in three fragments using 18S1F, 18S3F, 18S9R, 18S5R, 18Sbi, and 18Sa2.0 (Giribet et al., 1996; Whiting et al. 1997), resulting in sequence lengths up to 1927 bp. Up to 973 bp of 28S rRNA were amplified using Po28F1 and Po28R4 (Struck et al. 2006). Amplification was carried out with 12.5µl Apex 2.0x Taq RED DNA Polymerase Master Mix (Genesee Scientific), 1µl each of the appropriate forward and reverse primers (10µM), 8.5µl of ddH_2_O, and 2µl eluted DNA. The PCR reactions were carried out in a thermal cycler (Eppendorf). The COI temperature profile was as follows: 94 °C/180 s – (94 °C/30 s – 47 °C/45 s – 72 °C/60 s) * 5 cycles – (94 °C/30 s – 52 °C/45 s – 72 °C/60 s) * 30 cycles – 72 °C/300 s. The 16S temperature profile was as follows: 95 °C/180 s – (95 °C/40 s – 50 °C/40 s – 72 °C/50 s) * 35 cycles – 72 °C/300 s. The 18S1F/18S5R temperature profile was as follows: 95 °C/180 s – (95 °C/30 s – 50 °C/30 s – 72 °C/90 s) * 40 cycles – 72 °C/480 s. The 28S temperature profile was as follows: 95 °C/180 s – (95 °C/30 s – 55 °C/40 s – 72 °C/75 s) * 40 cycles – 72 °C/300 s. The PCR product was purified with the ExoSap-it protocol (USB, Affimetrix) and sequencing was performed by Eurofins Genomics (Louisville, KY).

### Phylogenetic analyses

Alignments of the newly generated sequences, along with sequence data from GenBank for the four genes presented in Table 1 and published in the most recent aphroditiform phylogeny (Zhang et al. 2018) were performed using MAFFT (Katoh and Standley 2013). Poorly-aligned regions of the three rDNA genes were removed using Gblocks v.0.91b (Catresana 2000), with least stringent settings. This resulted in two concatenated alignments, referred to here as complete and Gblocked. Maximum likelihood (ML) analyses were conducted on the two datasets using RaXML v.8.2.10 (Stamatakis 2014) with each partition assigned the GTR+G model. Node support was assessed via thorough bootstrapping (1000 replicates). Bayesian Inference (BI) analyses were also conducted using MrBayes v.3.2.6 (Rohnquist et al. 2012). Best-fit models for these partitions were selected using the Akaike information criterion (AIC) in jModelTest 2 (Darriba et al. 2012; Guindon and Gascuel 2003). Maximum parsimony (MP) analyses were conducted using PAUP* v.4.0a161 (Swofford 2002), using heuristic searches with the tree-bisection-reconnection branch-swapping algorithm and 100 random addition replicates. Support values were determined using 100 bootstrap replicates. The acoetid *Panthalisoerstedi* Kinberg, 1856, was selected as the outgroup based on recent phylogenomic analyses that place Acoetidae as the sister clade to Iphionidae (Zhang et al., 2018). Uncorrected pairwise distances were calculated for the COI dataset with PAUP* v.4.0a161 (Swofford 2002). Median-joining haplotype networks (Bandelt et al. 1999) for *Thermiphionerapanui* sp. n. and *T.fijiensis* were created with PopART v.1.7 (Leigh and Bryant 2015).

### Morphology

Most parsimonious reconstructions for a few relevant characters were mapped onto the molecular phylogeny of Iphionidae using Mesquite v.3.4 (Maddison and Maddison 2018). No DNA data is presently available for *Iphionellaphilippinensis*, or *Iphionidesglabra*, and they are not included in this study. Their eventual phylogenetic placement in Iphionidae will influence the inferred transformations found in this study. Morphological characters used were:

1. Elytra. Thirteen pairs of elytra are found in *Iphionella* (Pettibone, 1986), while *Thermiphione* has 14 pairs (Hartmann-Schröder 1992). Members of *Iphione* have 13 pairs of elytra (Pettibone 1986). The monotypic *Iphionides* has up to 20 pairs (Hartmann-Schröder 1977). Other Aphroditiformia, including the outgroup Acoetidae, normally have many elytral pairs. States, **0**. Many pairs; **1**. 13 pairs; **2**. 14 pairs.

2. Palps. Within Iphionidae, *Iphione* have papillate palps, while all other Iphionidae and the outgroup have smooth palps (Pettibone 1986, Gonzalez et al. 2018). States, **0**. Smooth; **1**. Papillate.

3. Eyes. Within Iphionidae, *Thermiphione* and *Iphionellarisensis* lack obvious eyes, while all other Iphionidae and the outgroup have them (Pettibone 1986, Gonzalez et al. 2018). States, **0**. Present; **1**. Absent.

4. Antennae. In general, Aphroditiformia have a median antenna, while most have lateral antennae (Gonzalez et al. 2018). Acoetidae have lateral and median antennae. A median antenna is absent in all Iphionidae, while the presence of lateral antennae varies. In *Iphione*, lateral antennae are present, while they are absent in *Iphionella*, *Iphionides* and *Thermiphione* (Pettibone 1986, Hartmann-Schröder 1992, Miura 1994). States, **0**. Present; **1**. Absent.

### Taxonomic note

*Iphionella* was erected by McIntosh (1885) as a new genus of Polynoidae for a specimen collected from ~900 meters depth from off Philippines, identified as *Iphionecimex* Quatrefages, 1866. This species was therefore the type species for *Iphionella* by monotypy. Pettibone (1986) determined that this identification by McIntosh as *Iphionecimex* was incorrect as the type of *Iphionecimex*, described from the Malacca Strait, actually belonged to Polynoidae and should be placed in a new genus, *Gaudichaudius* Pettibone, 1986, and so it was referred to as *G.cimex* (Quatrefages, 1866). Pettibone (1986) then redescribed the specimen McIntosh (1885) had used to erect *Iphionella* as a new species, *Iphionellaphilippinensis* Pettibone, 1986. This was not in accordance with the International Code on Zoological Nomenclature at the time (see Art. 70.3; ICZN, 1999). According to 70.3.1, the correct type species name for *Iphionella* was *Iphionecimex* Quatrefages, which should have become *Iphionellacimex* (Quatrefages, 1866). Furthermore, since *Iphionecimex* is the type species of *Gaudichaudia*, then *Gaudichaudia* should become a junior synonym of *Iphionella*. As a result of this, *Iphionella* should be referred to Polynoidae, and the two currently accepted species of *Iphionella*, *I.philippinensis* and *I.risensis* Pettibone, 1986 are in the incorrect genus and require new names. While technically correct, we regard this as not being in accordance of a goal of taxonomic nomenclature to provide stability of names. We therefore endorse Pettibone’s (1986) non-ICZN-compliant actions. In order to preserve stability, the type species of *Iphionella* is now fixed here (under Art. 70.3.2 of the ICZN) as *Iphionellaphilippinensis* Pettibone, 1986, misidentified as *Iphionecimex* in the original designation by McIntosh (1885).

## Results

**Figure 2.**
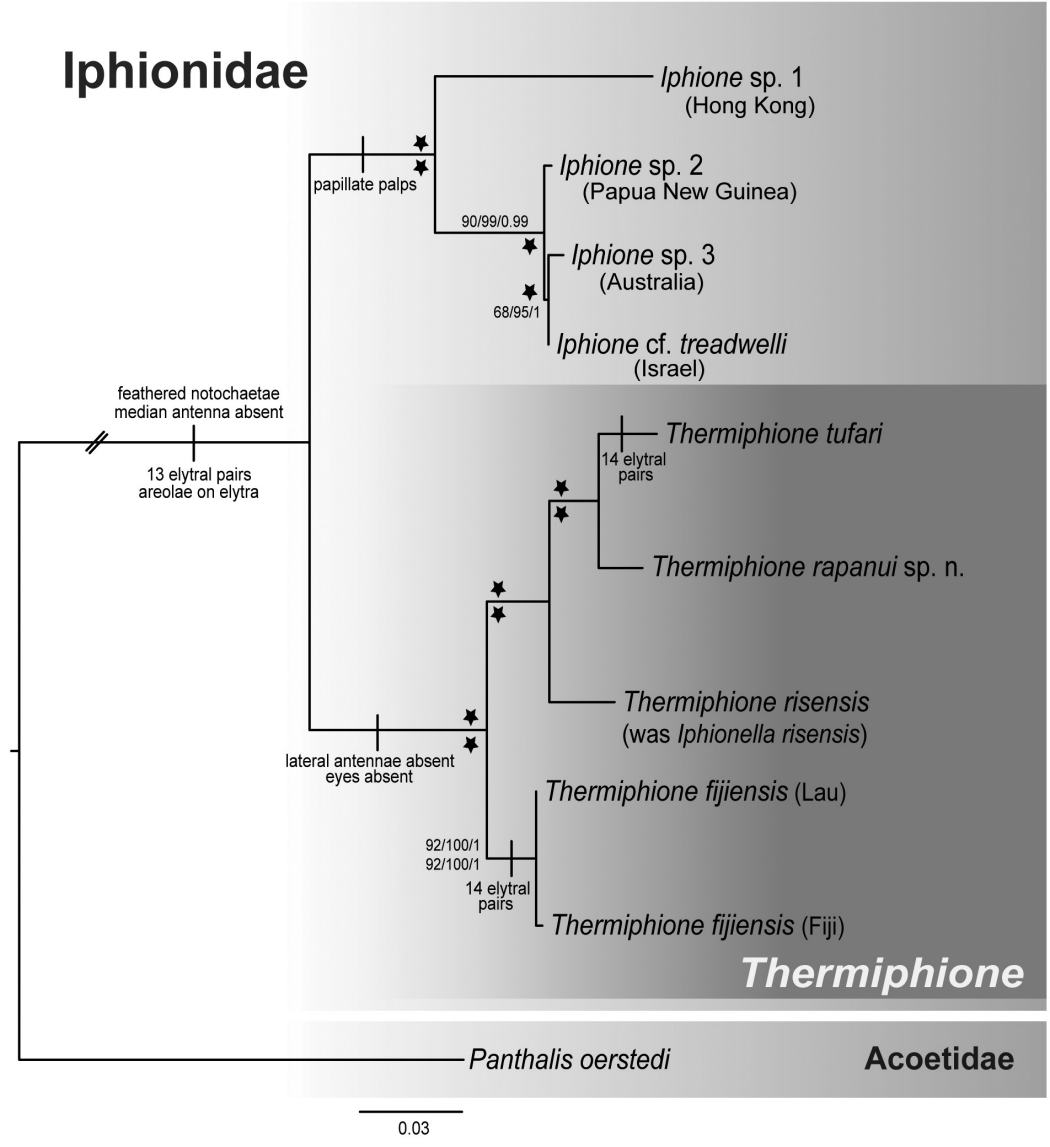
Maximum likelihood tree of the combined analysis from four genes (28S, 18S, 16S, COI) aligned with MAFFT and then concatenated (No Gblocks). Numbers above nodes are bootstrap support percentages from RAxML and Maximum Parsimony analyses (separated by slashes), followed by Bayesian posterior probabilities from the complete dataset alignment (no Gblocks) and below nodes from Gblocks. Support values of 95% or greater for all analyses are indicated by stars.

The complete and Gblocked ML, BI and MP analyses (Figure 2) were congruent, showing the same topology for relationships and generally similar high support values within Iphionidae (Figure 2), except for relationships within *Iphione*. The *Iphione* terminals formed a sister clade to a well-supported clade comprised of all the iphionids from hydrothermal vents.

The two known *Thermiphione* species, *T.fijiensis* and *T.tufari*, formed a grade with respect to *Iphionellarisensis* (Figure 2). The new species, *Thermiphionerapanui* sp. n., was the well-supported sister group to the sympatric *T.tufari*. The three East Pacific Rise taxa, *I.risensis*, *T.tufari* and *T.rapanui* sp. n. were recovered as the sister group to the western Pacific *T.fijiensis*. The taxonomic implications of the paraphyly of *Thermiphione* and our rationale for the generic placement of the new species are discussed below. The analysis of uncorrected pairwise COI distances (Table 3) showed that *T.rapanui* sp. n. was ~10.5% divergent from its sister taxon, *T.tufari*, and 13–15% divergent from *I.risensis* and *T.fijiensis* (Table 3). For the four specimens of *T.rapanui* sp. n. that we obtained COI sequences for there were three haplotypes that varied from each other by only two base pairs (Figure 4B).

**Table 3.** Uncorrected pairwise distances for COI data, generated with PAUP*.

	*Thermiphionerapanui* sp. n.	* Thermiphione tufari *	* Thermiphione fijiensis *	Thermiphione (Iphionella) risensis	* Iphione cf. treadwelli *	*Iphione* sp. 1	*Iphione* sp. 2
* Thermiphione tufari *	10.48%	–	–	–	–	–	–
* Thermiphione fijiensis *	15.39%	16.67%	–	–	–	–	–
Thermiphione (Iphionella) risensis	13.39%	14.25%	14.79%	–	–	–	–
Iphione cf. treadwelli	18.14%	19.88%	17.27%	19.23%	–	–	–
*Iphione* sp. 1	21.75%	19.73%	20.39%	21.52%	18.78%	–	–
*Iphione* sp. 2	23.81%	24.01%	21.66%	24.00%	23.35%	24.73%	–
*Iphione* sp. 3	18.49%	19.92%	17.42%	19.06%	0.76%	19.75%	23.14%

**Figure 3.**
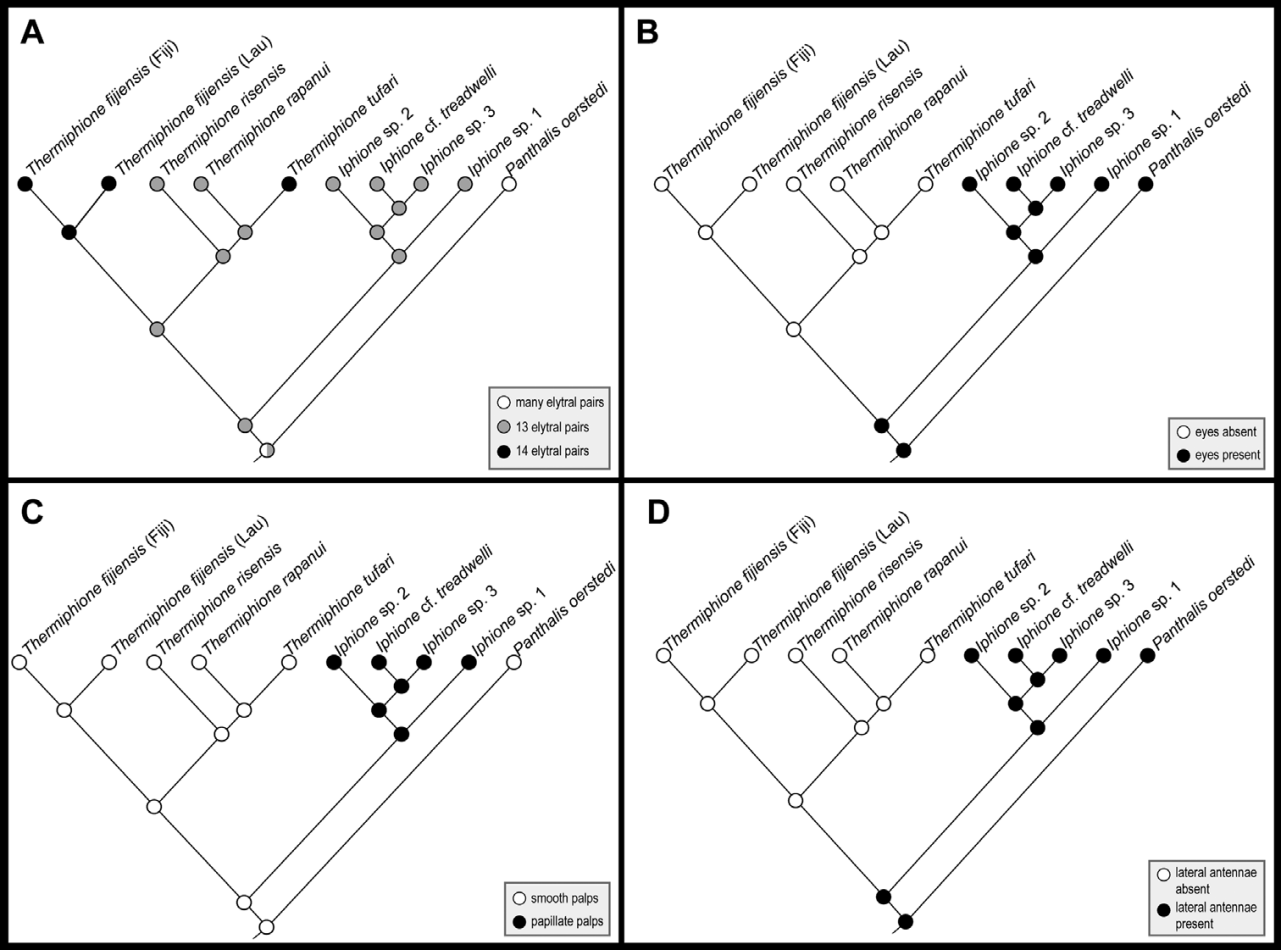
Most parsimonious reconstructions of four traits mapped onto the molecular phylogeny (complete dataset). **A** Elytral pairs **B** Eyes **C** Palps **D** Lateral antennae.

The parsimony reconstruction of ancestral states revealed an unambiguous convergent appearance of 14 pairs of elytra in *Thermiphionefijiensis* and *Thermiphionetufari* and that an elytral number of 13 represents the plesiomorphic state for Iphionidae. The absences of eyes and lateral antennae may be apomorphies for *Thermiphione* (but see below) (Figs 2, 3). The presence of papillate palps was apomorphic for *Iphione* (Figure 3).

**Figure 4.**
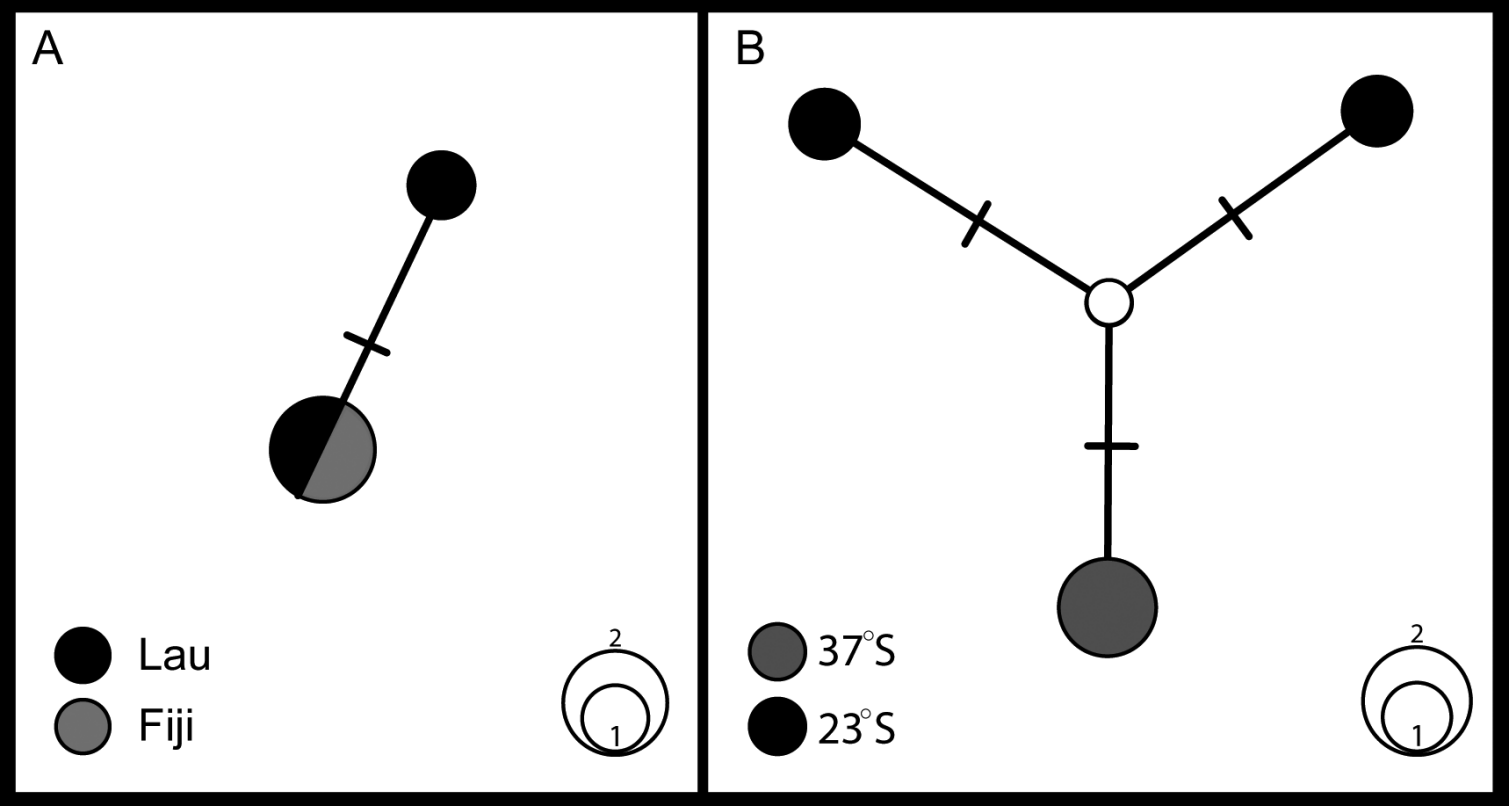
Haplotype networks from COI data: **A***Thermiphionefijiensis* network includes two sequences from specimens from the Lau Back-Arc Basin (black), and one from the type locality in Fiji (grey) **B***Thermiphionerapanui* sp. n. network includes two sequences from 23°S (black) and two from 37°S (grey).

### Taxonomy

#### Iphionidae Kinberg, 1856

##### Thermiphione

Taxon classificationAnimaliaPhyllodocidaIphionidae

Hartmann-Schröder, 1992, emended

http://zoobank.org/7BC3CE3F-4C9B-476A-A263-B8B77B961467

###### Type species.

***Thermiphionetufari* Hartmann-Schröder, 1992**

###### Diagnosis

**(emended).** Ventrally flattened, short, oval-shaped body. Between 28 and 32 segments in adults, with 13 or 14 pairs of elytra on segments 2, 4, 5, 7, 9, 11, 13, 15, 17, 19, 21, 23, 26 (and 27, if 14 pairs) that cover dorsal side. Elytra rounded, covered with polygonal and/or hexagonal areas with lattice-like areolae; may exhibit papillae along elytral margins and on elytral surface near margins. Bilobed prostomium square to oval, merged with segment 1, with short, smooth, bulbous palps. Lateral and median antennae absent. Eyes absent. Segment 1 with paired enlarged anterior cirri (*sensu*Rouse and Pleijel 2001; = tentacular cirri), bearing each pair on a tentaculophore with an acicula and capillary chaetae. Mouth anterior, not ventral. Eversible pharynx with papillae and two pairs of jaws. Segment 2 bears first pair of elytra and parapodia, spherical papillae. Segment 3 barely visible dorsally, with parapodia wedged between segments 2 and 4. Segments 4 and 7 bear spherical ventral papillae. All parapodia biramous: notopodia rounded and much smaller than neuropodia, with bundles of thin, feathered notochaetae; neuropodia large with thicker, single-tipped neurochaetae. Dorsal cirri with short papillae and cylindrical cirrophores. Ventral cirri much smaller than dorsal cirri, short and cirriform. Pygidium inconspicuous, lacking anal cirri.

###### Remarks.

**Figure 5.**
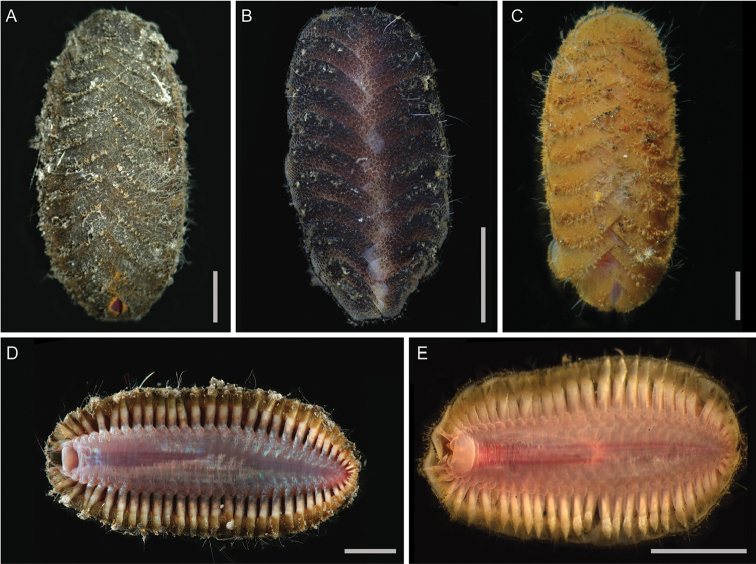
Dorsal and ventral micrographs of species in *Thermiphione*. Scale bars represent 5 mm. **A***Thermiphionefijiensis* (SIO-BIC A7975), dorsal **B***Thermiphionerisensis* (SIO-BIC A6326, was *Iphionellarisensis*), dorsal **C***Thermiphionetufari* (SIO-BIC A7973), dorsal **D***Thermiphionefijiensis* (SIO-BIC A7975), ventral **E***Thermiphionerisensis* (SIO-BIC A6326), ventral.

**Figure 6.**
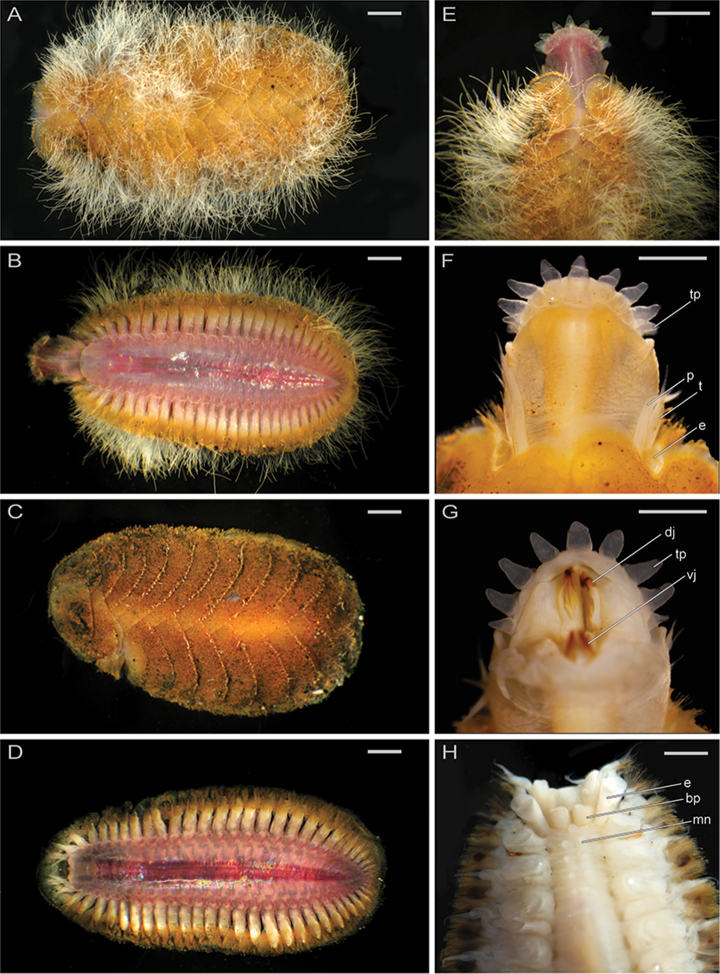
Micrographs of live *Thermiphionerapanui*, sp. n., holotype (SIO-BIC A8557) and paratype (SIO-BIC A7969). Scale bars in A–E represent 1 mm, and scale bars in F–H represent 0.5 mm. **A** Dorsal view of whole body, holotype **B** Ventral view of whole body with pharynx everted, holotype **C** Dorsal view of whole body, paratype **D** Ventral view of whole body, paratype **E** Dorsal view of anterior region with scales, holotype **F** Dorsal view of anterior region with 2 pairs of scales removed, holotype. Abbreviations as follows: *tp*, terminal papilla; *p*, palp; *t*, tentaculophore; *e*, elytrophore **G** Ventral view of anterior region with pharynx and jaws everted/visible, holotype. Abbreviations: *dj*, dorsal jaw; *tp*, terminal papilla; *vj*, ventral jaw **H** Dorsal view of anterior region, paratype. *e*, elytrophore; *bp*, prostomium (bilobed); *mn*, medial nodule.

**Figure 7.**
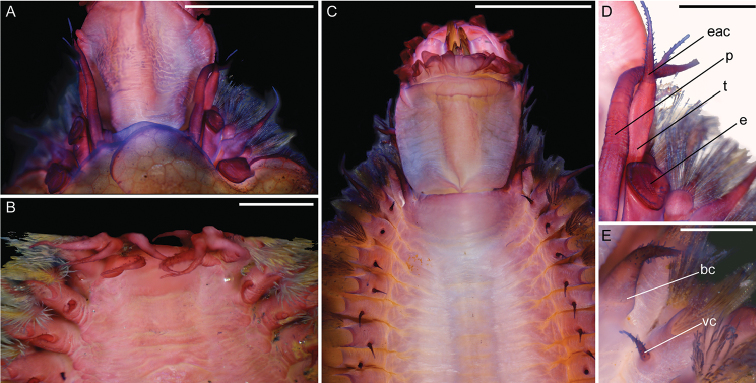
Micrographs of *Thermiphionerapanui* sp. n. holotype (SIO-BIC A8557) and paratype (SIO-BIC A7971), stained with Shirlastain-A. Scale bars in A–C represent 1 mm, and scale bars in D–E represent 0.25 mm. **A** Dorsal view of anterior with 2 pairs of scales removed, holotype **B** Ventral view of anterior showing palps tentaculophore and cirri, paratype. **C** Ventral view of anterior with pharynx everted and jaws visible, holotype **D** Magnified dorsal view of anterior right side, holotype. Abbreviations as follows: *e*, elytrophore; *p*, palp; *t*, tentaculophore; *eac*, enlarged anterior cirrus **E** Magnified ventral view of left anterior parapodia and ventral cirri on segments 2 and 3, holotype. Abbreviations: *bc*, buccal cirrus; *vc*, ventral cirrus.

**Figure 8.**
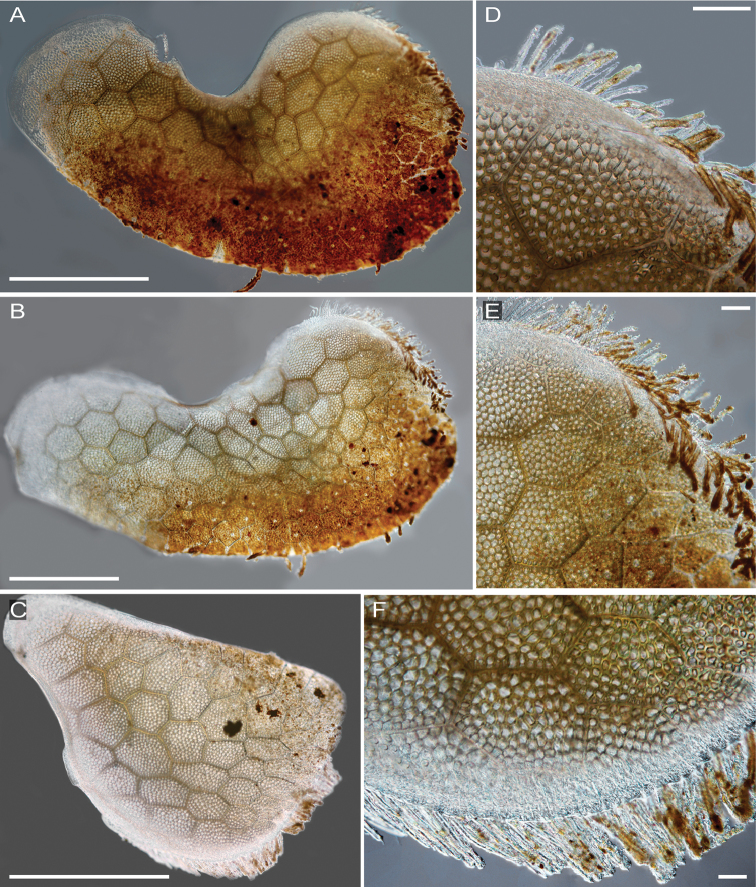
Interference contrast micrographs of *Thermiphionerapanui* sp. n elytra, paratype (SIO-BIC A7969). Scale bars in A–C represent 1mm, and scale bars in D–F represent 0.1 mm. **A** Right elytron 1 **B** Right elytron 3 **C** Left elytron 13 **D** Right elytron 1 margin **E** Right elytron 3 margin **F** Left elytron 13 margin.

**Figure 9.**
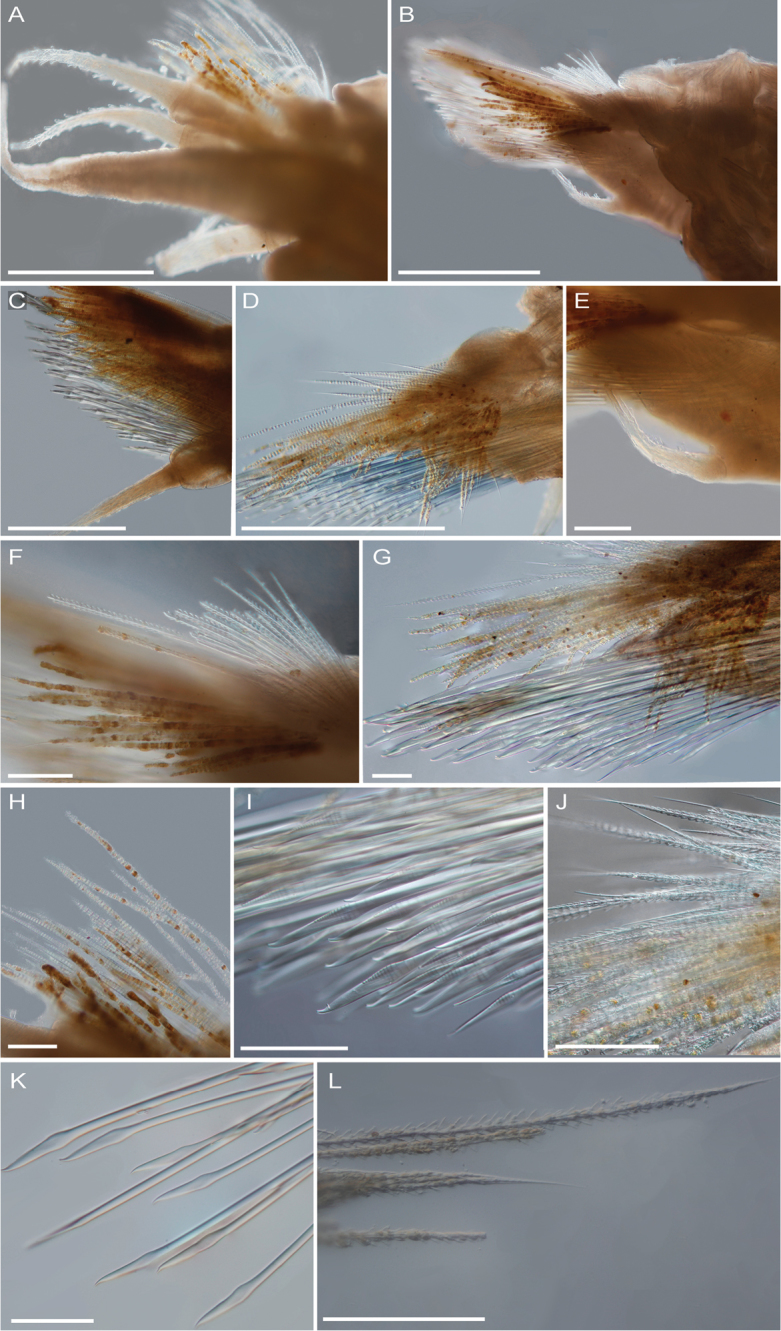
Interference contrast micrographs of *Thermiphionerapanui* sp. n. parapodia, (paratype SIO-BIC A7969). Scale bars in A–D represent 0.5 mm, and scale bars in E–L represent 0.1 mm. **A** Right parapodium 1 **B** Right parapodium 2 **C** Right parapodium 13 **D** Right parapodium 25 **E** Enlarged view of ventral cirrus (parapodium 2) **F** Feathered notochaetae (parapodium 2) **G** Chaetae of parapodium 25 **H** Notochaetae of right parapodium 2. **I** Slightly hooked neurochaetae (right parapodium 25) **J** Feathered notochaetae of parapodium 25 **K** Simple neurochaetae (some slightly hooked) from right parapodium 13. **L** Feathered notochaetae from right parapodium 13.

Hartmann-Schröder’s (1992) diagnosis of *Thermiphione* has been amended to accommodate the inclusion of *Iphionellarisensis* and *Thermiphionerapanui* sp. n. The genus now comprises *Thermiphionefijiensis* (Figure 5A, D), *T.risensis* (Figure 5B, E), *T.tufari* (Figure 5C), and *T.rapanui* sp. n (Figs 6–9). The morphology of these taxa and phylogenetic evidence suggests that segment and elytral numbers are more variable than in the previous diagnosis. *Thermiphione* all have smooth palps, but this is plesiomorphic for Iphionidae. The absence of eyes may be an apomorphic state, depending on the eventual placement of *Iphionellaphilippinensis*, which was not included here owing to the lack of material for DNA sequencing. Similarly, the loss of lateral antennae may also be an apomorphy for *Thermiphione* once the position of *Iphionellaphilippinensis* and *Iphionidesglabra*, which also lack them, is resolved.

##### Thermiphione rapanui sp. n.

Taxon classificationAnimaliaPhyllodocidaIphionidae

http://zoobank.org/D201192A-0569-4C3E-8B22-4C3C3C6A27D7

Figures 6 , 7 , 8 , 9

###### Type-locality.

German Flats, hydrothermal vents of Pacific Antarctic Ridge, 110°55'W, 37°48'S.

**Material Examined.***Type specimens.* Holotype (SIO-BIC A8557) from German Flats, hydrothermal vents of Pacific Antarctic Ridge, (type locality above), HOV *Alvin* Dive 4088, 2216m depth, 22 March 2005; fixed in 10% SW formalin, preserved in 50% ethanol. The holotype was not sequenced directly to avoid damage but was morphologically identical to sequenced specimens from the same locality. Post-preservation, holotype 10 mm long, 8.5 mm wide including parapodia, 31 segments. Paratypes: 1 specimen (SIO-BIC A7969) fixed and preserved in 95% ethanol, same location as holotype, post-preservation 9 mm long, 7 mm wide, 29 segments; 1 specimen (SIO-BIC A7970) from same location as holotype: anterior of specimen (approximately 14 segments) fixed in 10% SW formalin and preserved in 50% ethanol and posterior (approximately 14 segments) fixed and preserved in 95% ethanol; 2 specimens (SIO-BIC A7971, juvenile; SIO-BIC A7972) from the western flank of the Easter Microplate, East Pacific Rise, 115°34'W, 23°32'S, HOV *Alvin* Dive 4096, 2595m depth, 6 April 2005. SIO-BIC A7971 fixed and preserved in 95% ethanol, post-preservation 7 mm long, 4 mm wide, 19 segments; SIO-BIC A7972: anterior of specimen (approximately 20 segments) fixed in 10% SW formalin and preserved in 50% ethanol and posterior (approximately 9 segments) fixed and preserved in 95% ethanol.

###### Diagnosis.

Ventrally flattened, oval-shaped body. Between 29 and 31 segments, with 13 pairs of elytra on segments covering dorsum. Elytra covered completely by polygonal areas enclosing areolae, with marginal papillae covering edges. Prostomium bilobed and slightly rounded. Eyes absent. Lateral and median antennae absent. Segment 1 with pair of smooth palps and pair of tentaculophores plus enlarged anterior cirri (tentacular cirri). Mouth anterior with eversible pharynx. Segment 2 with buccal cirri. Segment 3 with dorsal tubercles. Dorsal cirri long with short styles. Ventral cirri short. Anus dorsal. Parapodia biramous with dense bundles of feathered notochaetae and less dense hooked neurochaetae.

###### Description.

In life, elytra pale brown with yellow tinge, becoming slightly paler after preservation. Body ventrally flattened, slightly tapered at anterior and posterior ends (Figure 6A–D). Holotype with 31 segments, 13 pairs of elytra, bacterial filaments on elytra (Figure 6A, B). One mature paratype SIO-BIC A7969, 29 segments, 13 pairs of elytra (Figure 6C, D). One juvenile paratype (SIO-BIC A7971), 19 segments, eight pairs of elytra (identified by scars; elytra lost in sampling).

Pharynx everted anteriorly in holotype, with 9 pairs terminal papillae, and dorsal and ventral pairs of hook-shaped jaws (Figs 6E–G, 7A–C). Prostomium bilobed, slightly rounded; eyes lacking (Figure 6H). Dorsal small circular medial nodules on segments 4 (1), and 5–8 (2 per segment) (Figure 6H). Lateral and median antennae lacking (Figs 6F–H, 7A–C). Pair of smooth palps, longer than pair of tentaculophores plus enlarged anterior cirri (tentacular cirri) (Figs 6F, 7A–B, D). Tentaculophores extending laterally to prostomium (Figs 6F, 7A–B, D), each with single acicula and very thin, short capillary chaetae on inner side. Enlarged anterior cirri, dorsal cirri, and ventral cirri with papillae (Figure 7). Buccal cirri on segment 2, also papillate, appearing larger than remaining ventral cirri (Figure 7C, E). Thirteen pairs of elytra covering dorsum and oval in shape, on segments 2, 4, 5, 7, 9, 11, 13, 15, 17, 19, 21, 23, 26 (Figure 8). First pair of elytra slightly compressed (Figure 8A); last pair much smaller in size and tapered at one end compared to other elytra (Figure 8B–C). Elytra covered completely by polygonal (generally hexagonal) areas enclosing areolae (Figure 8D–F). Thin, rounded marginal papillae covering lateral edges of elytra, sometimes sparsely extending towards posterior edges of elytra (Figure 8D-F). Remaining segments cirrigerous. Dorsal tubercles and dorsal cirri on segment 3, alternating on segments 6–29, with short, clavate papillae; anal cirri on segments 30, 31 (Figure 6B, D). Dorsal cirri long with short styles, usually extending to near tips of neurochaetae. Ventral cirri much shorter and smaller than dorsal cirri, present on segments 2–29 (Figure 7B–C, E). Anus dorsal; short ventral anal cirri similar to posterior dorsal cirri. Parapodia biramous (Figure 9), with short, subconical notopodia anterodorsal to larger neuropodia (Figure 9). Dense bundles of slender feathered notochaetae, shorter than neurochaetae (Figure 9F, H, J, L). Longer, simple, or slightly hooked neurochaetae, less dense but more numerous than notochaetae (Figure 9G, I, K). Upper neurochaetae generally longer than lower neurochaetae, with length of neurochaetae gradually decreasing towards dorsal and ventral edges (Figure 9).

*Variation.* Paratypes vary in segment number from holotype and were observed with fewer bacterial filaments on elytra.

###### Genetic distance.

Paratype specimens from the 23°S sampling locality varied by two nucleotide bases from the holotype specimen, 37°S (Figure 4B). This genetic distance is so small that they are certainly all the same species. Unfortunately, our sampling was too limited for any analyses of connectivity.

###### Etymology.

*Thermiphionerapanui* sp. n. is named after the traditional Polynesian name for Easter Island (Rapa Nui), which lies near one of the paratype localities. Neither of the specimens from near Easter Island were chosen as the holotype as they were in poor condition.

###### Remarks.

*Thermiphionerapanui* sp. n. was collected from hydrothermal vents across 15 degrees of latitude, with the northernmost samples collected from the western flank of the Easter Microplate region at 23°S latitude, and the samples from further south collected on the East Pacific Rise at 37°S. The northernmost samples of *Thermiphionerapanui* sp. n. were collected from the same locality as samples of its sister taxon, *T.tufari*, which previously has only been recorded from slightly further north at 21°30'S (Hartmann-Schröder 1992).

*Thermiphionerapanui* sp. n. differs from its sister taxon *T.tufari* in that it has 13 pairs of elytra instead of 14 pairs of elytra and the last pair of elytra are on segment 26 instead of segment 27 (compare dorsal photos of each in Figs 6A and 5C, respectively). Like *T.tufari*, the new species also has up to 31 segments (Hartmann-Schröder 1992). Both *T.tufari* and *T.fijiensis* (Figure 5A) have 14 pairs of elytra and 30–31 segments (Pettibone, 1986), so elytral number may be convergent (Figure 3). *Thermiphione* was erected by Hartmann-Schröder (1992) and distinguished from other Iphionidae largely based on the presence of 14 pairs of elytra and 30–31 segments, but *Iphionellarisensis* (Figure 5B), which nests within the *Thermiphione* (Figure 2), and *Thermiphionerapanui* sp. n. have 13 elytral pairs (Pettibone 1986). However, the two latter species differ in that *I.risensis* has 28–29 segments (Pettibone 1986) and *T.rapanui* sp. n. has 29–31 segments. *T.rapanui* sp. n. also differs from *I.risensis* in the presence of medial nodules on segments 6–8 in *T.rapanui* sp. n., which are absent on these segments in *I.risensis* (Pettibone 1986).

## Discussion

The topologies of the likelihood and parsimony phylogenies are similar to those recovered in the recent analyses of Norlinder et al. (2012), Gonzalez et al. (2018), and Zhang et al. (2018) and support the maintenance of Iphionidae as a family distinct from Polynoidae.

The phylogeny demonstrates that our newly generated sequences for *Thermiphionefijiensis* represent the same species as the *Thermiphione* sp. published in Norlinder et al. (2012). These specimens were collected on the same cruise as the Norlinder et al. (2012) specimen. The *Thermiphione* sp. (Norlinder) specimen was collected at the White Lady hydrothermal vent, near the type locality for *Thermiphionefijiensis*. It is therefore identified here as *T.fijiensis*. The two specimens of *Thermiphionefijiensis* collected from the Lau Back-Arc basin, varied at most by a single base pair from the Norlinder et al. (2012) sequences (Figure 4A).

The distribution of the three East Pacific Rise iphionids sampled in this study (Table 2) and the phylogenetic results (Figure 2) indicate that *Iphionellarisensis* forms a northern sister clade to the more southern *Thermiphionerapanui* sp. n. and *T.tufari* clades. This combined eastern Pacific clade is then sister group to *Thermiphionefijiensis* (Figure 2). The placement of *Iphionellarisensis* makes *Thermiphione*, as currently formulated, paraphyletic. To resolve the paraphyly of *Thermiphione, Iphionellarisensis* should be placed within *Thermiphione* and we do so here by amending the diagnosis for *Thermiphione* to allow for the presence of 13 or 14 pairs of elytra and 28–31 segments (see below). No DNA data currently exists for the type species of *Iphionella*, *I.philippinensis*.

## Supplementary Material

XML Treatment for Thermiphione

XML Treatment for Thermiphione rapanui
